# Real-world survival outcomes in patients with locally advanced or metastatic *NTRK* fusion-positive solid tumors receiving standard-of-care therapies other than targeted TRK inhibitors

**DOI:** 10.1371/journal.pone.0270571

**Published:** 2022-08-08

**Authors:** Derrek P. Hibar, George D. Demetri, Solange Peters, Jessica Davies, Olivier Humblet, Sophia L. Maund, Laura Perez

**Affiliations:** 1 Personalized Healthcare Analytics, Genentech, Inc., South San Francisco, California, United States of America; 2 Dana-Farber Cancer Institute and Ludwig Center at Harvard Medical School, Boston, Massachusetts, United States of America; 3 Lausanne University Hospital, Centre Hospitalier Universitaire Vaudois (CHUV), Lausanne, Switzerland; 4 Personalized Healthcare Data Science, Roche Products Ltd, Welwyn Garden City, United Kingdom; 5 Quantitative Sciences, Flatiron Health Inc., New York, New York, United States of America; 6 Oncology Biomarker Development, Genentech, Inc., South San Francisco, California, United States of America; 7 Personalized Healthcare Data Science, F. Hoffmann-La Roche Ltd, Basel, Switzerland; Qatar University College of Medicine, QATAR

## Abstract

The clinical profiles and outcomes of patients with neurotrophic tropomyosin receptor kinase fusion-positive (*NTRK*^+^) solid tumors receiving standard of care other than tropomyosin receptor kinase inhibitor (TRKi) targeted therapy have not been well documented. Here, we describe the clinical characteristics of patients with *NTRK*^+^ tumors treated in clinical practice using information from a United States electronic health record-derived clinicogenomic database. We also compared survival outcomes in *NTRK*^+^ patients and matched *NTRK* fusion-negative (*NTRK*^–^) patients and investigated the clinical prognostic value of *NTRK* fusions. *NTRK* positivity was defined by the presence of a fusion or rearrangement involving *NTRK1/2/3*, determined using NGS (Foundation Medicine, Inc.). *NTRK*^+^ patients (n = 28) were diagnosed with locally advanced/metastatic solid tumors between January 1, 2011 and December 31, 2019 and had received no TRKis (e.g., entrectinib or larotrectinib) during their patient journey. The unselected *NTRK*^−^population comprised 24,903 patients, and the matched *NTRK*^−^cohort included 280 patients. *NTRK*^+^ patients tended to be younger, were more commonly not smokers, and had a shorter time from advanced diagnosis to first NGS report, compared with unselected *NTRK*^−^patients; however, these differences were not significant. Median overall survival (OS) from advanced/metastatic diagnosis was 10.2 months (95% CI, 7.2–14.1) for the *NTRK*^+^ cohort versus 10.4 months (95% CI, 6.7–14.3) for the matched *NTRK*^−^cohort; hazard ratio for death in *NTRK*^+^ versus matched *NTRK*^−^patients was 1.6 (95% CI, 1.0–2.5; *P* = 0.05). Genomic co-alterations were rare in the *NTRK*^+^ cohort (only two of 28 patients had a co-alteration). Overall, while hazard ratios suggest *NTRK* fusions may be a negative prognostic factor of survival, there are no significant indications of any favorable impact of *NTRK* fusions on patient outcomes. TRKis, with their high response rate and good tolerability, are likely to improve outcomes for patients compared with existing standard-of-care treatments.

## Introduction

Fusions of any member of the neurotrophic tropomyosin receptor kinase (*NTRK1/2/3*) gene family can lead to expression of chimeric tropomyosin receptor kinase (TRK) transmembrane proteins with constitutively active kinase function [1]. *NTRK* gene fusions are validated oncogenic drivers and proven therapeutic targets across a range of tumor types, although the frequency of such fusions varies widely depending on tumor type (e.g., <5% in lung and colorectal cancers, 5–25% in thyroid cancer and >90% in mammary analogue secretory carcinoma) [2].

With the recent incorporation of TRK inhibitor therapies and molecular testing for *NTRK* gene fusions into clinical practice, it is important to assess whether there is any unique prognostic significance of *NTRK* gene fusions in order to put into context the efficacy noted with TRK-targeted therapies. The efficacy and safety of TRK inhibitors, such as larotrectinib and entrectinib, have been reported in both adults and children with *NTRK* fusion-positive (*NTRK*^+^) solid tumors, with objective response rates (ORR) of >65% and durable objective responses of >12 months [3–7]. These deep and durable responses contrast with the poor outcomes these patients anecdotally experienced on prior traditional standard-of-care (SoC) therapies. Consequently, regulatory authorities and the medical community have shown a growing interest in TRK-targeted therapies, illustrated by the tumor-agnostic approvals received by entrectinib and larotrectinib worldwide and the increasing number of TRK inhibitors currently in development. Importantly, when new drugs are evaluated in patients with rare tumor types or rare gene rearrangements such as *NTRK* fusions, the evidence packages submitted to health technology assessment (HTA) bodies are required to include sufficient evidence to allow assessment of any uniquely positive benefit of the new drug versus available SoC approaches. However, the clinical characteristics, treatment patterns and outcomes in patients with *NTRK*^+^ solid tumors under SoC therapies (e.g., chemotherapy, non-TRK inhibitor targeted therapy, immunotherapy, or hormonal therapy) are not well characterized and are possibly highly variable across cancer types. The rarity of these fusions means that relevant patient populations are small, and this limits the ability to perform randomized controlled trials. In addition, clinical trials for these rare biomarkers may include patients with varying and sometimes rare cancer types. This makes it difficult to design a study in which the efficacy of a TRK inhibitor would be directly compared to that of any single SoC. To overcome the challenges surrounding limited patient availability, outcomes from single-arm, tumor-agnostic clinical trials could be supported by real-world data, to better contribute to the robustness of drug filing applications and further improve their value to HTAs [8].

The objective of this study was to describe the clinical characteristics of patients with *NTRK*^*+*^ tumors treated in clinical practice with SoC therapies other than targeted TRK inhibitors, including testing patterns, demographics and overall survival (OS), using information from a large clinicogenomic database. Furthermore, the potential for any clinical prognostic value of *NTRK* fusions was evaluated by comparing outcomes in patients harboring such fusions (*NTRK*^*+*^ patients) with outcomes in matched patients with solid tumors that do not harbor such fusions (*NTRK*^*−*^patients).

## Study design

This is a retrospective analysis of clinical characteristics and survival outcomes of patients with *NTRK*^*+*^ tumors treated in clinical practice, using information from the Flatiron Health–Foundation Medicine de-identified clinicogenomic database (FH-FMI CGDB), a US-wide longitudinal database curated through technology-enabled abstraction. Data from patients diagnosed with a locally advanced/metastatic solid tumor from January 1, 2011 to December 31, 2019 were selected. The primary outcome assessments used time-to-event analyses for two index times, which correspond to the date of diagnosis of advanced/metastatic disease (main analysis), and the start date of the last available treatment line before next generation sequencing (NGS) test report (sensitivity analysis), respectively. The sensitivity analysis was done to account for interpatient differences in number of prior treatment lines and time before they had any actionable NGS result.

## Patients and methods

### Ethics statement

This study used de-identified patient data from the Flatiron Health–Foundation Medicine clinicogenomic database (FH-FMI CGDB), a US-wide longitudinal database curated through technology-enabled abstraction, and did not directly enroll patients.

### Data source

The FH-FMI CGDB includes patients from a subset of the Flatiron Health network of ~280 United States (US) cancer clinics (approximately 800 care sites); the majority of patients within the database originate from community oncology settings. Retrospective longitudinal clinical data were derived from electronic health record data, comprising patient-level structured and unstructured data, curated through technology-enabled abstraction and linked to genomic data derived from FMI comprehensive genomic profiling tests in the FH-FMI CGDB by de-identified, deterministic matching. Data from the FH-FMI CGDB were collected from routine healthcare practice, and all patient tumors had undergone comprehensive genomic profiling at Foundation Medicine. The database allows aggregation and processing of patient-level data such as demographics, diagnostic information (e.g., stage; pathology; molecular information; radiology), extent of disease, laboratory findings, treatments (e.g., line of therapy; dosing; regimens), and patient outcomes.

### Population

Patients included in the *NTRK*^+^ population had tumor tissue tested with ≥1 DNA and/or RNA-based NGS assay (Foundation Medicine, Inc. [FMI]), at the clinician’s discretion, and had ≥1 *NTRK*^+^ test result. Other inclusion criteria for the *NTRK*^+^ study population were: diagnosis of a locally advanced (not amenable to radical therapy)/metastatic solid tumor from January 1, 2011 to December 31, 2019; age ≥18 years; no prior treatment with entrectinib or larotrectinib in any therapy line (patients could have received other anti-cancer therapies); no visit gap of >90 days after diagnosis of locally advanced/metastatic disease (this was to exclude patients who may have been treated temporarily in a non-Flatiron Health network center post-diagnosis); no prior unlabeled study drug as part of a clinical trial. *NTRK*-positivity was defined by the presence of a fusion or rearrangement involving *NTRK1/2/3* with predicted known/likely functional status as defined by FMI; fusion calls with non-protein coding gene partners or intragenic fusions were excluded [9]. By contrast, an *NTRK*^−^status was determined when an NGS test with on-panel *NTRK* was unable to detect qualifying *NTRK* fusions.

### Objectives

#### Clinical parameters

Demographic and clinical data were collected by mining the Flatiron Health data. Information provided at the date of diagnosis or at the most recent previous visit or encounter was used to describe patient demographics and clinical characteristics at baseline. Variables and categories used in this study included sex, age group, ethnicity, tumor histology, tumor stage at initial diagnosis, smoking status, Eastern Cooperative Oncology Group performance status (ECOG PS), practice type (academic center or community setting), time from diagnosis to reported NGS test date, presence of central nervous system (CNS) disease, and number of treatment lines.

#### Outcomes

The primary outcome for this study was OS, defined as the length of time in months from the index date (advanced/metastatic disease diagnostic) until death from any cause or the censoring date (i.e., last visit or encounter date). Date of death was a composite endpoint based on an algorithm linking patient-level electronic health record-derived data to an external commercial mortality data source and the US Social Security Administration’s Death Index [10, 11].

### Statistical analyses

#### Descriptive analyses

Descriptive statistics were used to assess baseline characteristics, testing patterns, and presence/absence of other relevant molecular alterations in patients with *NTRK*^+^ solid tumors.

#### Overall survival

The OS assessments used time-to-event analyses for two index times derived for the *NTRK*^+^ and the matched *NTRK*^*−*^cohorts. For each index time, we estimated median OS from Kaplan-Meier curves and estimated differences in prognosis using univariate Cox proportional hazards models for the *NTRK*^+^ and the matched *NTRK*^*−*^cohorts. To provide additional benchmarks for survival from diagnosis to death, OS Kaplan-Meier curves and summary statistics were also derived for the overall unselected (non-matched) *NTRK*^−^population from the FH-FMI CGDB. All estimations accounted for differences in times of patient entry to cohort via risk set adjustment, so as to minimize the immortal bias dependent on reported NGS test results.

#### Prognosis assessment

The prognostic value of the *NTRK* fusion biomarker was evaluated using a univariate Cox proportional hazard model comparing OS in the *NTRK*^+^ cohort to that of the matched *NTRK*^*−*^cohort, with median results presented with the hazard ratio (HR), 95% confidence interval (CI), and *P-*values. The matched *NTRK*^*−*^cohort was developed using the nearest neighbor propensity score (PrS) matching model based on the tumor types observed in the *NTRK*^+^ cohort (Table 1). To minimize overfitting the model because of small sample size, the PrS was developed by logistic regression on the basis of a minimum a priori set of selected prognostic variables (i.e., age, smoking status, practice type, number of lines of antineoplastic treatments since initial diagnosis, stage at initial diagnosis, reported time between locally advanced/metastatic diagnosis and reported test). In addition, co-mutations were included in the PrS, since selected co-biomarkers or genetic alterations that are known actionable targets or proxies for other driver mutations can be used as prognostic factors and linked to treatment decisions and outcomes. In particular, high tumor mutational burden (TMB-H) and high microsatellite instability (MSI-H) are important in predicting response to certain therapies [12–14]. To avoid false positives, a conservative TMB-H was defined as ≥20 mutations per Mb and low TMB as <6 mutations per Mb. Based on current treatment guidelines and giving priority to targets for which a recommended specific drug exists [15, 16], selected co-alterations included *ALK* fusions, *ROS1* fusions, *RET* fusions, *EGFR* L858R mutation, *EGFR* acquired T790M mutation, *EGFR* exon19 deletion, *BRAF* V600E mutation, *BRAF* V600K mutation, *MET* exon14 mutation, *KRAS* G12 mutation, and *KRAS* G13 mutation. As several of these genetic alterations occur in multiple tumors, they were all simultaneously considered in each tumor type. The ECOG PS was not included as a variable because of the high rate of unknown performance statuses in the database, thereby preventing imputation given the small number of patients in the *NTRK*^+^ cohort. For each prognostic factor included in the PrS, missing or unknown values were considered a separate variable category.

**Table 1:** Frequency of the tumor types found in the *NTRK*^+^ cohort in the three FH-FMI CGDB cohorts of interest.

Tumor type / location, n (%)	*NTRK*^−^FH-FMI CGDB (unselected)	*NTRK*^−^FH-FMI CGDB (matched)	*NTRK*^*+*^ FH-FMI CGDB
	N = 24,903	N = 280	N = 28
**Colorectal cancer**	4,197 (16.9)	90 (32.1)	9 (32.1)
**Soft tissue sarcoma**	281 (1.1)	60 (21.4)	6 (21.4)
**Non-small cell lung cancer**	6,064 (24.4)	50 (17.9)	5 (17.9)
**Salivary gland**	37 (0.1)	20 (7.1)	2 (7.1)
**Breast**	2,969 (11.9)	10 (3.6)	1 (3.6)
**Cancer of unknown primary**	1,477 (5.9)	10 (3.6)	1 (3.6)
**Stomach**	516 (2.1)	10 (3.6)	1 (3.6)
**Bile duct**	286 (1.1)	10 (3.6)	1 (3.6)
**Endometrium**	237 (1.0)	10 (3.6)	1 (3.6)
**Uterus**	52 (0.2)	10 (3.6)	1 (3.6)

Abbreviations: FH-FMI CGDB, Flatiron Health–Foundation Medicine clinicogenomic database; *NTRK*^-^, neurotrophic tropomyosin receptor kinase fusion-negative; *NTRK*^+^, neurotrophic tropomyosin receptor kinase fusion-positive.

The unselected *NTRK*^−^FH-FMI CGDB cohort includes other tumor types that are not represented in the *NTRK*^+^ cohort and thus not included in this table.

A matching ratio of one patient with an *NTRK*^+^ tumor to 10 patients with *NTRK*^*−*^tumors was used in the main analyses. The characteristics of the *NTRK*^+^ cohort and the matched *NTRK*^*−*^cohort (Table 2) were compared via standardized mean differences (SMD). The SMD was also used to evaluate the balance between the cohorts: an SMD >0.2 (>20% difference) or *P*-value <0.05 after matching was indicative of a difference between groups (described previously [17, 18]). Kaplan-Meier curves and summary statistics (i.e., median OS with 95% CI) are also presented for the matched and unselected *NTRK*^−^cohorts.

**Table 2:** Comparison of patient characteristics for the unselected *NTRK*^–^, matched *NTRK*^−^and *NTRK*^*+*^ cohorts.

	Level	*NTRK*^−^FH-FMI CGDB (unselected)	*NTRK*^*−*^FH-FMI CGDB (matched)	*NTRK*^*+*^ FH-FMI CGDB	*P* *	SMD*
**N**		**24,903**	**280**	**28**		
**Age category, n (%)**	18–34	497 (2.0)	0 (0.0)	0 (0.0)	0.785	0.094
	35–64	12,062 (48.4)	157 (56.1)	17 (60.7)		
	≥65	12,344 (49.6)	123 (43.9)	11 (39.3)		
**Gender, n (%)**	Female	12,648 (50.8)	131 (46.8)	18 (64.3)	0.117	0.358
	Male	12,255 (49.2)	149 (53.2)	10 (35.7)		
**Smoking status, n (%)**	History of smoking	14,511 (58.3)	127 (45.4)	12 (42.9)	0.957	0.05
	No history of smoking	10,076 (40.5)	153 (54.6)	16 (57.1)		
	Unknown/not documented	316 (1.3)	0 (0.0)	0 (0.0)		
**Practice type, n (%)**	Academic	2,829 (11.4)	53 (18.9)	4 (14.3)	0.728	0.125
	Community	22,074 (88.6)	227 (81.1)	24 (85.7)		
**ECOG PS, n (%)**	0	4,940 (19.8)	58 (20.7)	5 (17.9)	0.883	0.318
	1	7,449 (29.9)	68 (24.3)	9 (32.1)		
	2	2,285 (9.2)	22 (7.9)	2 (7.1)		
	3	675 (2.7)	8 (2.9)	0 (0.0)		
	4	56 (0.2)	2 (0.7)	0 (0.0)		
	Unknown	9,498 (38.1)	122 (43.6)	12 (42.9)		
**Number of prior lines of treatment (%)**	None	2,902 (11.7)	21 (7.5)	1 (3.6)	0.929	0.239
	1	9,683 (38.9)	108 (38.6)	10 (35.7)		
	2	4,361 (17.5)	67 (23.9)	9 (32.1)		
	3	2,273 (9.1)	20 (7.1)	2 (7.1)		
	4	1,077 (4.3)	9 (3.2)	1 (3.6)		
	5+	1,469 (5.9)	0 (0.0)	0 (0.0)		
	Unknown	3,138 (12.6)	55 (19.6)	5 (17.9)		
**Stage at initial diagnosis, n (%)**	Stage 0	10 (0.0)	0 (0.0)	0 (0.0)	0.977	0.14
	Stage I	1,434 (5.8)	26 (9.3)	3 (10.7)		
	Stage II	2,986 (12.0)	21 (7.5)	2 (7.1)		
	Stage III	4,381 (17.6)	54 (19.3)	4 (14.3)		
	Stage IV	13,561 (54.5)	133 (47.5)	14 (50.0)		
	Unknown	2,531 (10.2)	46 (16.4)	5 (17.9)		
**CNS metastases, n (%)**	No/unknown	22,252 (89.4)	267 (95.4)	23 (82.1)	0.016	0.428
	Yes	2,651 (10.6)	13 (4.6)	5 (17.9)		
**Time from advanced/ metastatic diagnosis to test, mean (SD)**		272.10 (446.92)	158.95 (371.16)	151.21 (245.20)	0.914	0.025
**Year of reported test, n (%)**	2012	29 (0.1)	2 (0.7)	0 (0.0)	0.967	0.373
	2013	400 (1.6)	10 (3.6)	1 (3.6)		
	2014	1,591 (6.4)	21 (7.5)	3 (10.7)		
	2015	2,513 (10.1)	50 (17.9)	4 (14.3)		
	2016	2,967 (11.9)	34 (12.1)	4 (14.3)		
	2017	4,264 (17.1)	50 (17.9)	4 (14.3)		
	2018	5,535 (22.2)	52 (18.6)	6 (21.4)		
	2019	6,336 (25.4)	50 (17.9)	6 (21.4)		
	2020	1,268 (5.1)	11 (3.9)	0 (0.0)		

Abbreviations: CNS, central nervous system; ECOG PS, Eastern Cooperative Oncology Group performance status; FH-FMI CGDB, Flatiron Health–Foundation Medicine clinicogenomic database; *NTRK*^-^, neurotrophic tropomyosin receptor kinase fusion-negative; *NTRK*^+^, neurotrophic tropomyosin receptor kinase fusion-positive; SMD, standardized mean difference; SD, standard deviation.

*For *NTRK*^*+*^ versus matched *NTRK*^−^populations.

#### Sensitivity analyses

The robustness of the main analyses in assessing the prognosis of the *NTRK*^+^ cohort was tested using different matching ratios and evaluating differences between the results. An alternative model was also conducted to match patients in the *NTRK*^+^ cohort with *NTRK*^−^control patients; contrary to the main analysis these were matched by all covariates except tumor type. Finally, selected co-alterations prior to PrS matching were restricted in the main analysis (i.e., excluding patients with any of the selected co-alterations) of the *NTRK*^*+*^ and *NTRK*^−^cohorts; a matching model allowing for co-alterations in both cohorts was also subsequently derived. This allowed us to evaluate the impact of including co-alterations in the prognosis value, but also to represent the gap in treatment benefit that may exist between patients with the *NTRK* biomarker and other alterations for which some targeted therapies already exist. The start of last available line before test report was chosen for this sensitivity analysis because it represents a decision point just before receiving NGS results, however this index remains close [average ~40 days] to the time of advanced diagnosis (used as index for the main analysis).

## Results

### Characteristics of patients in the NTRK+ cohort

**Fig 1:**
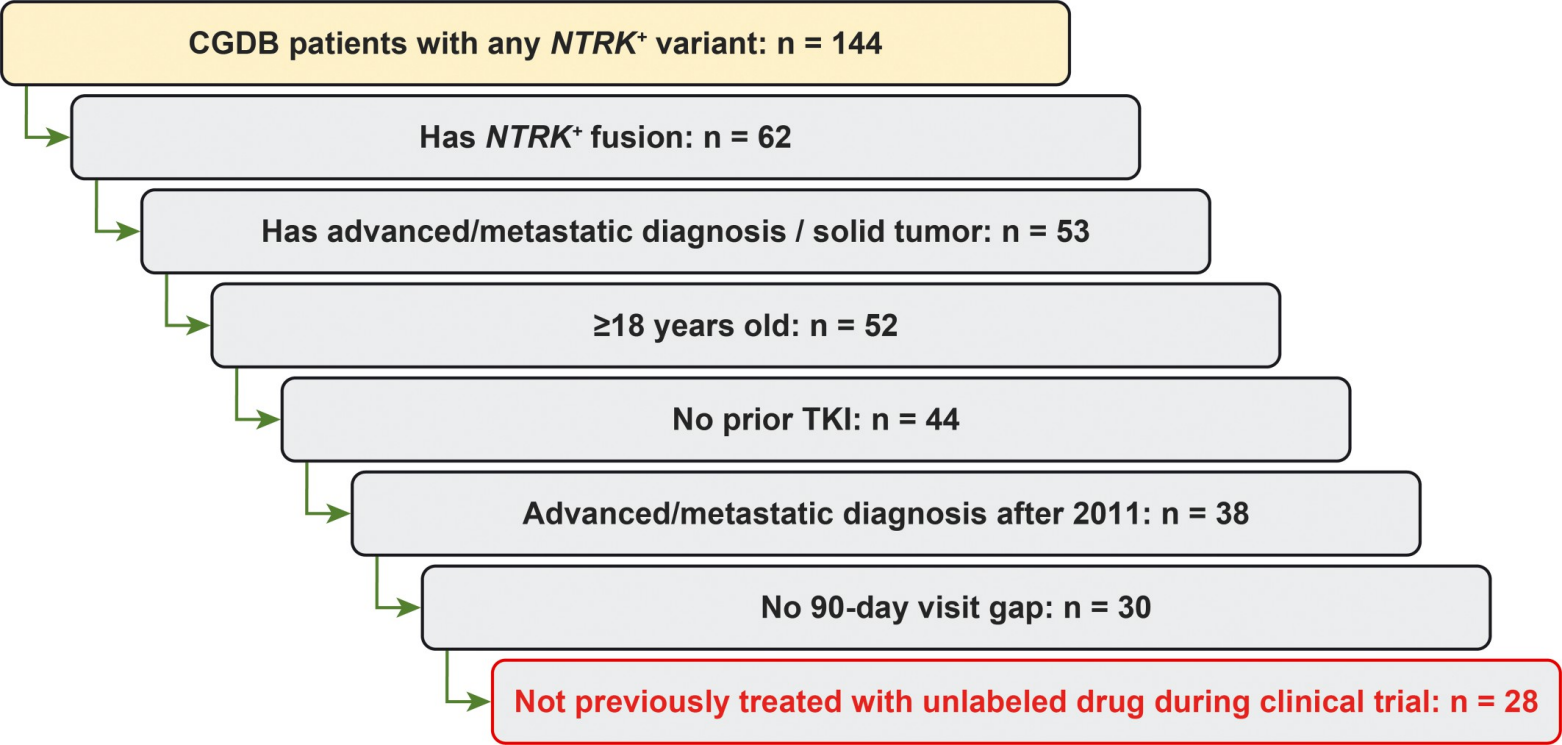
Cohort attrition. Abbreviations: CGDB, clinicogenomic database; NTRK^+^, neurotrophic tropomyosin receptor kinase fusion-positive; TKI, tyrosine kinase inhibitor.

Out of 58,001 patients within the database, 46,943 had solid tumors (cut-off: December 31, 2019), including 52 adult patients with a diagnosis of locally advanced/metastatic disease (0.11%) had an *NTRK*^+^ solid tumor (Fig 1). Twenty-four patients were excluded; eight had previously been treated with a TRK inhibitor (all with larotrectinib, one had also received entrectinib), eight had a visit gap since advanced/metastatic diagnosis of >90 days, six had a diagnosis of advanced/metastatic disease made before 2011, and two had received an unlabeled drug during a clinical trial. Therefore, the evaluable *NTRK*^+^ cohort included 28 patients (0.06% of patients with solid tumors from the database). The unselected *NTRK*^*−*^population comprised 24,903 patients who also met the above criteria (53.0% of patients with solid tumors from the database), while the matched *NTRK*^−^cohort consisted of 280 patients. In the *NTRK*^+^ cohort (n = 28), 10 different cancer types were identified; the most common were colorectal cancer (32%), soft-tissue sarcoma (21%), and non-small cell lung cancer (NSCLC; 18%; Table 1). Other tumor types were only identified in one or two patients each.

Twenty-three of the 28 *NTRK*^+^ patients (82%) had a fusion with *NTRK1;* the most common *NTRK1* fusion partners were *TPM3* (26%) and *LMNA* (22%) (S1 Table). The mean (standard deviation [SD]) time from diagnosis of locally advanced/metastatic disease to reported NGS test result was 151.21 (245.20) days (S1 Fig). A reported time of >50 days (i.e., an average first cycle of therapy) from advanced/metastatic diagnosis to testing was observed across various tumor types, with only four patients (colorectal cancer, n = 3; squamous cell carcinoma of the lung, n = 1) tested before this diagnosis. Most patients within the *NTRK*^+^ cohort were women (64%), 35–64 years of age (61%), and did not have a smoking history (57%; Table 2).

Information on the clinical characteristics of the *NTRK*^+^ cohort was missing for many patients: for example, 18% lacked information on disease stage at initial diagnosis; 43% had missing ECOG PS; 82% had no or unknown CNS metastases status (no distinction is made between no CNS metastases vs. unknown CNS metastases status in the CGDB). Of the patients with available data (i.e., excluding those with “unknown” status), 61% (n = 14/23) of patients were diagnosed with stage IV disease at initial diagnosis, and 88% (n = 14/16) had an ECOG PS of 0–1. Due to the limited data available for ECOG PS and CNS metastases, these two characteristics were not used in further modeling. Information on treatment patterns was also missing for 5 (18%) patients (Table 2). Across all lines of therapy in patients with available data, the most frequent treatment types were chemotherapy and chemotherapy in combination with targeted therapy (S2 Table). These two treatment types combined represented 91% of first-line therapies, 83% of second-line therapies, 33% of third-line therapies, and 0% of fourth-line therapies. S3 Table presents the number of lines of therapy received by the *NTRK*^+^ population, by tumor type. Most patients had received 1–2 lines of therapy before the index date, except for one patient with colorectal carcinoma, one patient with sarcoma and one patient with breast cancer who had received several successive treatment lines.

Genomic co-alterations were rare in the *NTRK*^+^ cohort (S4 Table). Within the available NGS test panel, one patient with *NTRK*^+^ sarcoma also presented an *EGFR* L858R mutation, and one patient with *NTRK*^*+*^ NSCLC presented a *KRAS* G12 mutation. Only 17 out of 28 (61%) patients had available data for MSI status; five of these 17 patients (29%), were categorized as MSI-H. Similarly, TMB status was not available for 12 out of 28 patients (43%); six of the patients who had available data (38%) were classified as TMB-H.

### Comparison of patient characteristics between the NTRK+ cohort and unmatched or matched NTRK–populations

Compared with the unselected *NTRK*^*−*^cohort, patients in the *NTRK*^+^ cohort tended to be younger, were more commonly not smokers (no smoking history: 57% in the *NTRK*^+^ cohort vs. 41% in the unselected *NTRK*^−^cohort), and had a shorter time from diagnosis of locally advanced/metastatic disease to a reported test (mean 151 vs. 272 days; Table 2). Interestingly, when looking at a subset of lung cancer patients, all (n = 5/5) *NTRK*^+^ patients had a history of smoking, compared with 83% (n = 5,045/6,065) of the unselected *NTRK*^*−*^patients (S5 Table). No differences were statistically significant. Unselected *NTRK*^*−*^patients had mostly received 1–2 prior treatment lines (Table 2; S6 Table), regardless of tumor type, suggesting that testing usually occurred after 1–2 lines of treatment, and not at initial diagnosis. Differences in tumor type proportions across cohorts (Table 1) may have contributed to the observed differences in co-mutation prevalence (S4 Table). MSI-H and TMB-H were markedly less prevalent in the *NTRK*^−^cohort versus the *NTRK*^+^ cohort (S4 Table).

Variables were generally balanced (no significant differences based on p-values) between the *NTRK*^+^ and the matched *NTRK*^*−*^cohorts (Table 2; S4 Table). Similar to the *NTRK*^+^ cohort, matched *NTRK*^*−*^patients had mostly received 1–2 prior treatment lines, had a time from advanced/metastatic diagnosis to test of ~150 days (mean 159 days vs. 151), and more than half had no history of smoking. While no patients from the *NTRK*^+^ cohort had an ECOG PS >2 at baseline, 10 out of 280 patients (3.6%) from the matched *NTRK*^−^population had an ECOG PS of 3 or 4. Since matching included the presence/absence of selected co-alterations and MSI-H and TMB-H, the prevalence of *KRAS* mutations, high TMB and high MSI was similar between the matched *NTRK*^−^and *NTRK*^+^ cohorts (S4 Table).

### Overall survival

**Fig 2:**
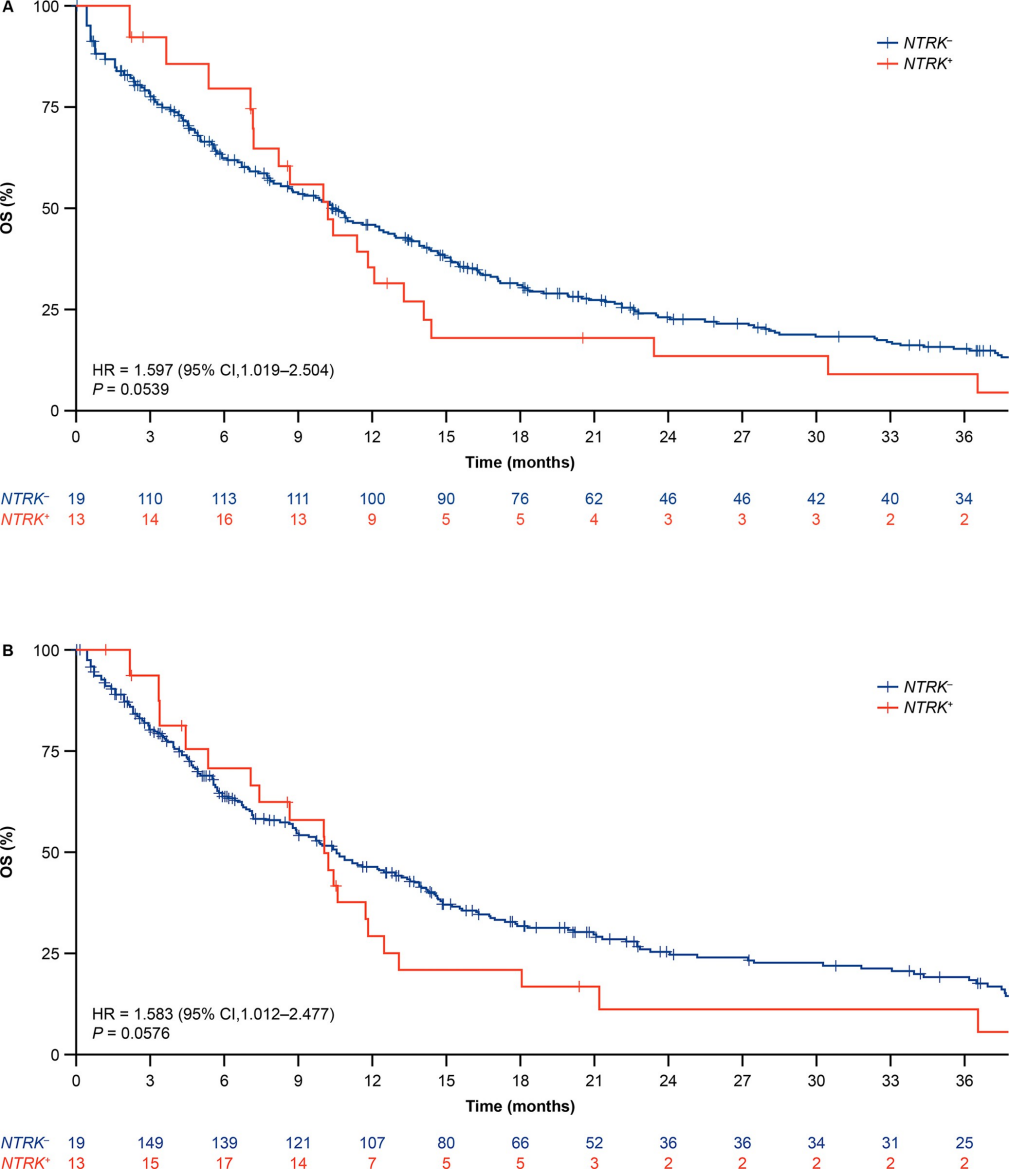
Kaplan-Meier curve of overall survival for the *NTRK*^+^ versus matched *NTRK*^−^cohorts using (A) index date as the date of diagnosis of locally advanced/metastatic disease adjusted for left truncation and (B) index date as the start date of the last treatment line before NGS test report, or locally advanced/metastatic disease if no line of therapy received and adjusted for left truncation. Abbreviations: CI, confidence interval; HR, hazard ratio; NGS, next generation sequencing; *NTRK*^-^, neurotrophic tropomyosin receptor kinase fusion-negative; *NTRK*^+^, neurotrophic tropomyosin receptor kinase fusion-positive; OS, overall survival. Due to immortal time bias and delayed entry, not all patients entered the risk set at t0.

In the *NTRK*^+^ cohort, the median OS from diagnosis was rather poor at 10.2 (95% CI, 7.2–14.1) months (Table 3). Using a matching ratio of 1:10, the median OS (that accounts for differences in cohort entry time) was similar in the matched *NTRK–*cohort (10.4 months [95% CI, 6.7–14.3]) and numerically (overlapping reference intervals) shorter in the unselected *NTRK–*cohort (9.0 months [95% CI, 8.7–9.3]). The HR for death in *NTRK*^+^ versus matched *NTRK*^*−*^(HR = 1.6; 95% CI, 1.0–2.5; *P* = 0.05; Fig 2A) was not statistically significant.

**Table 3:** OS analysis by cohort (date of diagnosis of locally advanced/metastatic disease used as index).

	Median follow-up time (months)	N	No. of events	% of events	Median OS (months)	95% CI
**Unselected *NTRK*** ^**−**^ **CGDB**	12.6	24,903	14,563	58%	9.0	8.7–9.3
**Matched *NTRK*** ^**−**^ **CGDB**	13.9	280	164	59%	10.4	6.7–14.3
***NTRK***^**+**^ **FH-FMI CGDB**	10.3	28	22	79%	10.2	7.2–14.1

Abbreviations: CI, confidence interval; FH-FMI CGDB, Flatiron Health–Foundation Medicine clinicogenomic database; *NTRK*^–^, neurotrophic tropomyosin receptor kinase fusion-negative; *NTRK*^+^, neurotrophic tropomyosin receptor kinase fusion-positive; OS, overall survival.

Results were similar if the start of the last treatment line before NGS test report was used as the index (HR = 1.6; 95% CI, 1.0–2.5; *P* = 0.06 [Fig 2B]). In this analysis, the median OS was similar to that presented from diagnosis (*NTRK*^+^ cohort: 10.1 [95% CI, 7.1–13.1] months; matched *NTRK*^−^cohort: 10.5 [95% CI, 8.6–13.9]; unselected *NTRK*^−^cohort: 8.9 [95% CI, 8.6–9.1] months). Due to the small sample size in the *NTRK*^+^ cohort, overall survival estimation per tumor type was only conducted in the unselected *NTRK*^*−*^cohort (S2 Fig). As might be expected by differences across cancer types, considerably different patterns of survival were seen depending on tumor type. Only patients with colorectal cancer had high median OS of >12 months; patients with breast, endometrial, stomach or salivary gland tumors, sarcoma or NSCLC showed a moderate OS of 7–12 months and those with uterine or biliary cancers, or CUP had a poor OS of <7 months. Importantly, the *NTRK*^−^cohort was not enriched for tumor types with shorter survival (S7 Table), suggesting that the median OS observed in this population was not expected to be biased by tumor biology.

### Sensitivity analyses

Reanalysis of the prognosis for patients in the *NTRK*^+^ cohort using matching ratios from 1:1 to 1:10, showed model stabilization starting after a minimum ratio of 1:6, and appearing to fully stabilize at a ratio of 1:10 (Table 4). When patient matching was done regardless of the co-alteration profile, median OS for the *NTRK*^−^cohort was numerically shorter than in the main analysis (9.3 months; 95% CI, 5.8–12.4) but the HR point estimate was not substantially changed (HR = 1.5; 95% CI, 1.0–2.4; *P* = 0.08 [sensitivity analyses 3 in Table 4]). Using a more restrictive matching (i.e., excluding patients harboring any of the selected co-alterations), median OS was slightly improved at ~9.9 months (95% CI, 6.3–13.8). The HR point estimate for this sensitivity analysis remained unchanged (HR = 1.5; 95% CI, 1.0–2.5; *P* = 0.08 [sensitivity analyses 4 in Table 4]).

**Table 4:** Comparison of prognosis results with different matching modeling approaches.

Model	Description	HR *NTRK*^*+*^ versus matched *NTRK*^–^(95% CI)	*P*-value
**Main model**	By tumor type, match includes presence/absence of selected co-alterations and MSI-H and TMB-H, matching ratio 1:10	1.597 (1.019–2.504)	0.0539
**Sensitivity analyses 1**	As main model, line of therapy definition from metastatic diagnosis until reported test (instead of initial diagnosis)	1.583 (1.012–2.477)	0.0576
**Sensitivity analyses 2**	As main model, matching ratio 1:6	1.609 (1.004–2.577)	0.0594
**Sensitivity analyses 3**	By tumor type, co-alteration in controls allowed, matching ratio 1:10	1.527 (0.975–2.393)	0.0793
**Sensitivity analyses 4**	By tumor type, a priori restricted unselected *NTRK*^*−*^with no co-alterations, includes MSI-H and TMB-H matching, matching ratio 1:10	1.549 (0.968–2.479)	0.0838
**Sensitivity analyses 5**	No match by tumor type, match includes presence/absence of co-alterations and MSI-H and TMB-H, matching ratio 1:10	1.398 (0.892–2.189)	0.161

Abbreviations: HR, hazard ratio; MSI-H, microsatellite instability–High; TMB-H tumor mutational burden–High; *NTRK*^–^, neurotrophic tropomyosin receptor kinase fusion-negative; *NTRK*^+^, neurotrophic tropomyosin receptor kinase fusion-positive

## Discussion

This analysis described the clinical characteristics and survival of TRK-inhibitor-naïve patients with *NTRK*^*+*^ tumors previously treated with SoC therapies other than targeted TRK inhibitors. A median OS of 10.2 months was observed in the small cohort of 28 patients with *NTRK*^*+*^ tumors, and a similar overall median OS across tumors (10.4 months) was observed in matched patients with *NTRK*^−^tumors (n = 280; HR = 1.6 [95% CI, 1.0–2.5; *P* = 0.05]). Due to the small sample size of the *NTRK*^*+*^ cohort, the study had limited power to detect a significant difference. Longer OS has been reported in recent clinical trials assessing the efficacy of TRK-targeted therapies (e.g., entrectinib and larotrectinib) in patients with *NTRK*^+^ tumors. For instance, in an integrated analysis of three phase 1/2 clinical trials of the TRK inhibitor entrectinib in 121 patients with *NTRK*^+^ tumors, median OS was 33.8 months (95% CI, 23.4–46.4) [5, 19].

Indirect comparisons between data from clinical trials of TRK inhibitors and the real-world assessment presented here support the hypothesis that OS in patients with *NTRK*^+^ solid tumors may be considerably improved by treatment with TRK inhibitors versus the current SoC, supporting the need for broader testing of targetable oncogenic biomarkers and the selection of the most appropriate therapy based on the tumor’s molecular profile. Notably, this comparison must be made with caution as there are no direct comparisons between the different therapies from head-to-head trials. OS was selected as the only clinical outcome in this study because it is often considered the most objective and best efficacy outcome to represent long-term clinical benefit for cancer therapies, when evaluating management approaches, including regulatory approval and reimbursement decisions [20]. However, the composite endpoint of OS has certain challenges, including applicability for tumors with relatively long survival as there may be large censoring, and crossover to new highly effective treatment strategies upon accessibility (confounding any possible significant differences in OS which otherwise might have occurred without access). Conversely, this outcome is well curated and studied in the FH-FMI CGDB and is likely to have limited bias compared with real-world assessments of progression-free survival or ORR, which are highly dependent on subjective measures (e.g., by investigator assessment).

Interestingly, another retrospective study that was conducted prior to the approvals of TRK inhibitor therapies also examined real-world outcomes in patients with *NTRK*^*+*^ solid tumors treated with SoC [21]. Using a somewhat different set of data from the FH-FMI CGDB, Bazhenova et al. reported a median OS of 12.5 months (95% CI, 9.5–not estimable) in 27 patients with *NTRK*^+^ solid tumors and 16.5 months (95% CI, 12.5–22.5) in 107 matched patients with *NTRK*^−^tumors. The *NTRK*^+^ cohort had a median age of 60 (range 49–65) and was 55% female. The most common primary tumors were colorectal cancer (24%), salivary gland cancer (17%), lung cancer (14%) and sarcoma (14%). ECOG PS data were missing for over 80% of the patients in this analysis; all patients with available data (17%) had ECOG PS 0–1.

A number of key differences between the two analyses can be noted. Firstly, while both studies involved patients with *NTRK*^+^ solid tumors from the FH-FMI CGDB, we selected locally advanced/metastatic disease as an inclusion criterion to align with the populations from the clinical trials; disease stage was not an inclusion criterion in the study by Bazhenova et al., suggesting that patients with early-stage disease (known to have a better prognosis [22]) may have also been included. This may also be the reason why the patient population from Bazhenova et al.’s study included some tumor types not found in our cohort (e.g., thyroid and pancreatic cancers). Another difference between the two populations is that our study excluded patients treated with any unlabeled clinical trial therapy or TRK inhibitor. Although Bazhenova et al. conducted their study prior to the approval of larotrectinib and entrectinib in the US, one patient with *NTRK*^+^ disease had received an unknown investigational agent in a clinical trial.

Other differences in methodology between the two studies bear further examination to better integrate both sets of findings. Our study included FH-FMI CGDB data from January 1, 2011 to December 31, 2019 compared with January 1, 2011 to July 31, 2018 (prior to the larotrectinib and entrectinib approvals in the US) for the study by Bazhenova et al.; this led to us having a considerably larger cohort of patients from which to obtain case/control pairs (46,943 vs. 33,429 patients with solid tumors; 24,903 vs. 12,456 *NTRK*^−^unselected patients). Interestingly, despite these differences, Bazhenova et al. reported a similar number of *NTRK*^+^ patients to our study (29 and 28, respectively). Considering that populations in observational studies such as these are very heterogeneous, a larger pool of *NTRK*^−^cases from which to obtain suitable matches for *NTRK*^*+*^ patients may be advantageous.

Overall, despite differences in methodologies the two studies showed similar OS HRs: Bazhenova et al. reported an OS HR of 1.4 (95% CI, 0.61–3.37; *P* = 0.648), while the OS HR in our study was 1.6 (95% CI, 1.0–2.5; *P* = 0.05). Taken together, these data do not suggest any favorable impact of *NTRK* fusions on responses to standard treatments or on survival outcomes, although it is difficult to draw definitive conclusions due to the small sample size of datasets currently existing in these rare biomarker populations. Additionally, patients with *NTRK* fusions may benefit from targeted therapies that have been shown in clinical trials to generally be more tolerable and efficacious than many SoC options.

Our study has several limitations. Except for using the matching ratio, our prognostic model was insensitive to alternative matching models. While we included a-priori variables known to be related to prognosis, and considered variables that impact treatment decisions in this specific setting of differential NGS testing, it is not certain that all differences were captured. In addition, although generalization is limited given the small *NTRK*^+^ patient numbers, the proportion of patients with MSI-H tumors seemed noticeably higher in the *NTRK*^*+*^ cohort (17.9%) compared with the unmatched *NTRK*^−^cohort (1.3%): this is in line with a previous report from Gatalica et al. [23] that suggested *NTRK*^+^ tumors are more likely to also be MSI-H. Considering MSI-H is known to affect responses to certain anti-cancer therapies [12–14], and that drugs with long-lasting outcomes are also approved for the MSI-H indication, real-world data could have an important role when evaluating the outcomes of patients with *NTRK* fusions and high MSI treated with immunotherapy, in order to help make the most informed treatment decision.

Furthermore, our study population was extracted from mostly routine care, where NGS testing is not yet spontaneously carried out for some cancer types. It is also not possible to evaluate whether the patients included in this study are a biased sample of all patients with *NTRK*^+^ disease. However, the prevalence of tumors detected in this study does not resemble the distribution observed among patients with *NTRK*^*+*^ tumors in the FoundationCORE database of >290,000 cases [24] (most common tumor types: NSCLC [16%], breast cancer [14%] and sarcoma [10%]). This suggests a possible bias due to only certain tumor types getting tested in community oncology centers within the Flatiron Health network, although we cannot draw definitive conclusions due to the small number of *NTRK*^+^ tumors in the FH-FMI CGDB.

Our study population also did not capture patients tested with NGS tests other than those from FMI. Patients treated in centers that use FMI testing may be different from patients treated in centers that use other NGS methods. Therefore, we may not be able to extrapolate these results to more generalized populations of patients with *NTRK*^+^ tumors. In addition, FMI assays do not cover the entire exome/genome, so while all exons and selected introns of *NTRK1/2/3* are baited, it is possible that some *NTRK* fusions may have been missed. Finally, a bias may have been introduced as the NGS test may have been selectively performed on patients who did not respond to SoC treatments. Additionally, we could not match all relevant confounders and prognostic factors, such as ECOG PS, given the limitations of the database. However, on the variables matched in this analysis, a good balance between cohorts was obtained, suggesting minimal unmeasured bias.

## Conclusion

Overall, our study shows that, while the hazard ratios suggest a trend for these fusions to potentially be a negative prognostic factor, the interpretation of the results is limited because of the small sample size; outcomes should be validated by repeating analyses in different and larger datasets when feasible. The relatively short OS observed in this real-world cohort of patients provides more evidence that SoC therapies have only a limited activity and that the reported efficacy of TRK inhibitors, such as entrectinib, is unique and clinically meaningful in patients with *NTRK*^+^ tumors.

## Supporting information

S1 Fig Time from advanced/metastatic diagnosis to reported test for the 10 tumor types observed in the *NTRK*^+^ cohort. Abbreviations: FMI, Foundation Medicine, Inc.; *NTRK*^+^, neurotrophic tropomyosin receptor kinase fusion positive.
(TIF)

S2 Fig Estimated overall survival in the unselected *NTRK*^−^cohort based on the tumor types observed in the *NTRK*^+^ cohort. Abbreviations: *NTRK*^-^, neurotrophic tropomyosin receptor kinase fusion negative; *NTRK*^+^, neurotrophic tropomyosin receptor kinase fusion positive.
(TIF)

S1 Table Gene fusion partners in the *NTRK*^*+*^ cohort. Abbreviations: *NTRK*^+^, neurotrophic tropomyosin receptor kinase fusion positive.
(DOCX)

S2 Table Distribution of treatment types by line of treatment received by patients in the *NTRK*^+^ cohort, between initial diagnosis and NGS test report. Abbreviations: NGS, next generation sequencing; *NTRK*^+^, neurotrophic tropomyosin receptor kinase fusion positive. *One patient had an *NTRK*^*+*^ test before starting treatment (so not included in this table), and treatment information was not available for five patients (so not included in this table).
(DOCX)

S3 Table Number of lines of therapy received by tumor type in the *NTRK*^+^ cohort. Abbreviations: *NTRK*^+^, neurotrophic tropomyosin receptor kinase fusion positive.
(DOCX)

S4 Table Comparison of selected biomarkers and genetic alteration status for the unselected *NTRK*^–^, matched *NTRK*^−^and *NTRK*^*+*^ cohorts. Abbreviations: FH-FMI CGDB, Flatiron Health–Foundation Medicine clinicogenomic database; MSI, microsatellite instability; MSI-H, high MSI; MSI-I, intermediate MSI; MSS, microsatellite stability; NA, not available; *NTRK*^-^, neurotrophic tropomyosin receptor kinase fusion negative; *NTRK*^+^, neurotrophic tropomyosin receptor kinase fusion positive; SMD, standardized mean difference; TMB, tumor mutational burden. *For *NTRK*^*+*^ versus matched *NTRK*^−^population.
(DOCX)

S5 Table Smoking status in patients with lung cancer from the overall, unselected *NTRK*^−^and *NTRK*^+^ cohorts. Abbreviations: FH-FMI CGDB, Flatiron Health–Foundation Medicine clinicogenomic database; *NTRK*^-^, neurotrophic tropomyosin receptor kinase fusion negative; *NTRK*^+^, neurotrophic tropomyosin receptor kinase fusion positive.
(DOCX)

S6 Table Number of lines of therapy received by tumor type/location in the unselected FH-FMI CGDB *NTRK*^*−*^cohort. Abbreviations: CNS, central nervous system; FH-FMI CGDB, Flatiron Health–Foundation Medicine clinicogenomic database; GI, gastrointestinal; GIST, gastrointestinal stromal tumor; neuro, neuroendocrine; NK T cells, natural killer T cells; *NTRK*^-^, neurotrophic tropomyosin receptor kinase fusion negative. The shades of orange represent the three largest groups in each tumor type (darker shades show the largest groups).
(DOCX)

S7 Table Overall survival per tumor type in the unselected *NTRK*^*−*^cohort. Abbreviations: CNS, central nervous system; FH-FMI CGDB, Flatiron Health–Foundation Medicine clinicogenomic database; GI, gastrointestinal; GIST, gastrointestinal stromal tumor; neuro, neuroendocrine; NK T cells, natural killer T cells; *NTRK*^-^, neurotrophic tropomyosin receptor kinase fusion negative; *NTRK*^+^, neurotrophic tropomyosin receptor kinase fusion positive; OS, overall survival. Green: tumor types with median OS >10 months; Red: tumor types with median OS <5 months; Gray: tumor types with unavailable median OS.
(DOCX)
