# Supplementary figures and images for: Real-world survival outcomes in patients with locally advanced or metastatic NTRK fusion-positive solid tumors receiving standard-of-care therapies other than targeted TRK inhibitors
Source: PLoS One. 2022 Aug 8;17(8):e0270571. doi: 10.1371/journal.pone.0270571 (PMC9359555; doi:10.1371/journal.pone.0270571)

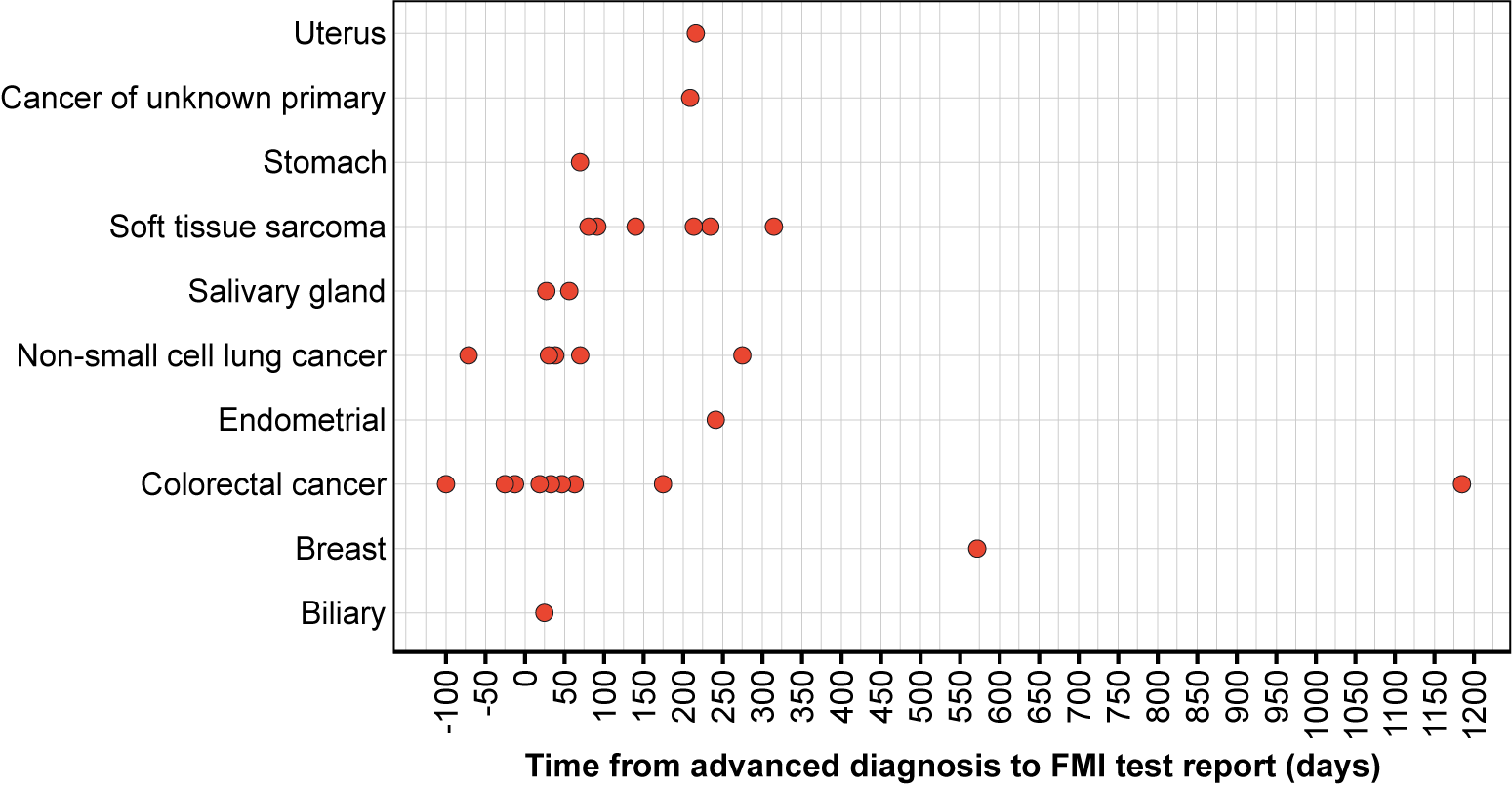

Supplement: S1 Fig — Abbreviations: FMI, Foundation Medicine, Inc.; NTRK+, neurotrophic tropomyosin receptor kinase fusion positive. (TIF) [file pone.0270571.s001.tif]

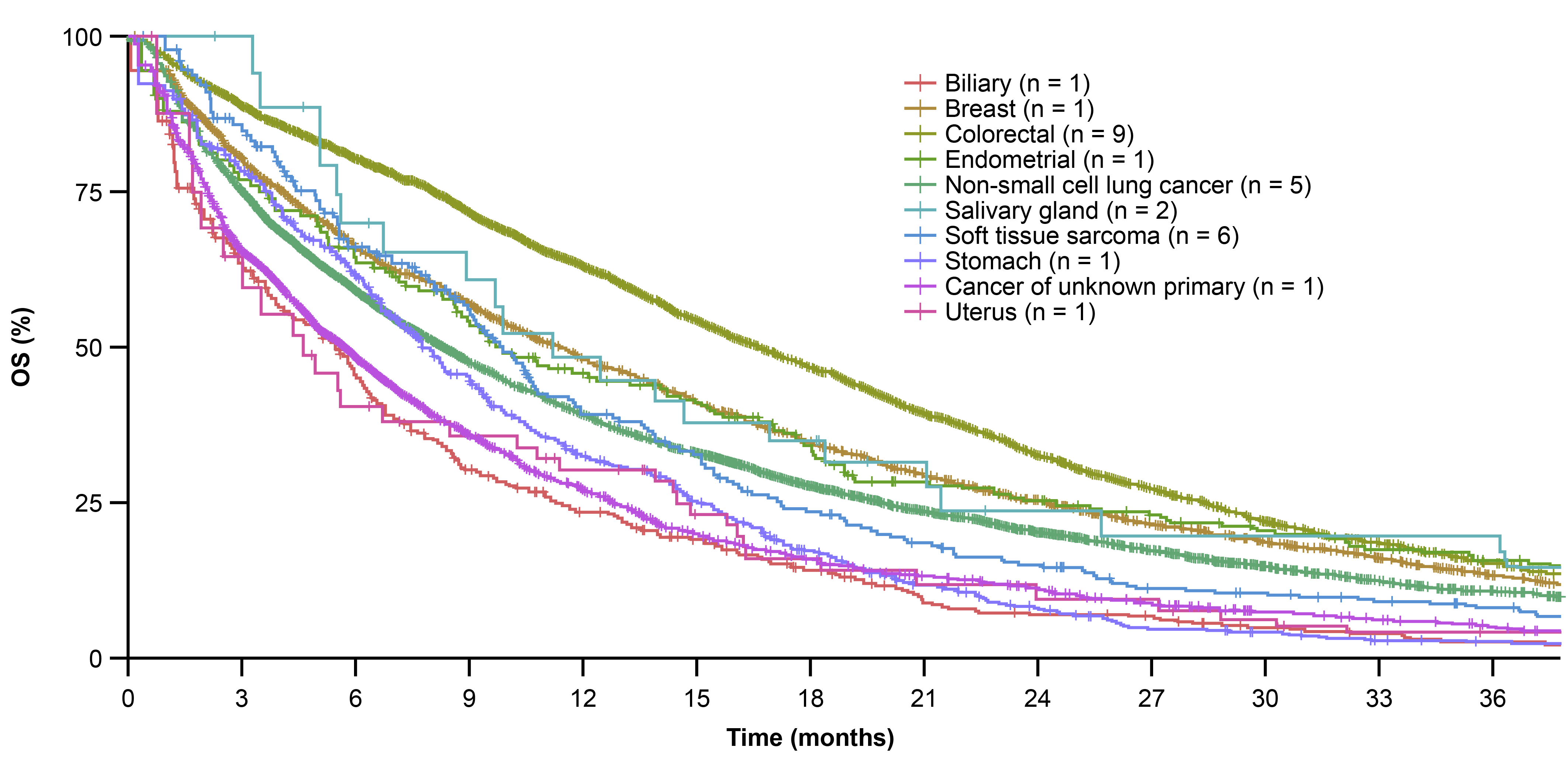

Supplement: S2 Fig — Abbreviations: NTRK-, neurotrophic tropomyosin receptor kinase fusion negative; NTRK+, neurotrophic tropomyosin receptor kinase fusion positive. (TIF) [file pone.0270571.s002.tif]
