# Supplementary material for: Real-world survival outcomes in patients with locally advanced or metastatic NTRK fusion-positive solid tumors receiving standard-of-care therapies other than targeted TRK inhibitors
Source: PLoS One. 2022 Aug 8;17(8):e0270571. doi: 10.1371/journal.pone.0270571 (PMC9359555; doi:10.1371/journal.pone.0270571)
Supplement: S1 Table — Abbreviations: NTRK+, neurotrophic tropomyosin receptor kinase fusion positive. (DOCX) [file pone.0270571.s003.docx]

| **n (%)** | ***NTRK1*** | ***NTRK2*** | ***NTRK3*** | **Overall** |
| --- | --- | --- | --- | --- |
|  | **23** | **2** | **3** | **28** |
| ***TPM3*** | 6 (26.1) | 0 | 0 | 6 (21.4) |
| ***LMNA*** | 5 (21.7) | 0 | 0 | 5 (17.9) |
| ***ETV6*** | 0 | 0 | 2 (66.7) | 2 (7.1) |
| ***MEF2D*** | 1 (4.3) | 0 | 0 | 1 (3.6) |
| ***PEAR1*** | 1 (4.3) | 0 | 0 | 1 (3.6) |
| ***PLEKHA6*** | 1 (4.3) | 0 | 0 | 1 (3.6) |
| ***RCSD1*** | 1 (4.3) | 0 | 0 | 1 (3.6) |
| ***SLC25A44*** | 1 (4.3) | 0 | 0 | 1 (3.6) |
| ***TPM1*** | 1 (4.3) | 0 | 0 | 1 (3.6) |
| ***TPR*** | 1 (4.3) | 0 | 0 | 1 (3.6) |
| ***VPS13D*** | 1 (4.3) | 0 | 0 | 1 (3.6) |
| ***FLVCR1*** | 1 (4.3) | 0 | 0 | 1 (3.6) |
| ***GRIPAP1*** | 1 (4.3) | 0 | 0 | 1 (3.6) |
| ***IRF2BP2*** | 1 (4.3) | 0 | 0 | 1 (3.6) |
| ***JAK1*** | 1 (4.3) | 0 | 0 | 1 (3.6) |
| ***ACO1*** | 0 | 1 (50.0) | 0 | 1 (3.6) |
| ***FRMD5*** | 0 | 1 (50.0) | 0 | 1 (3.6) |
| ***HOMER2*** | 0 | 0 | 1 (33.3) | 1 (3.6) |
