# Supplementary material for: Real-world survival outcomes in patients with locally advanced or metastatic NTRK fusion-positive solid tumors receiving standard-of-care therapies other than targeted TRK inhibitors
Source: PLoS One. 2022 Aug 8;17(8):e0270571. doi: 10.1371/journal.pone.0270571 (PMC9359555; doi:10.1371/journal.pone.0270571)
Supplement: S2 Table — Abbreviations: NGS, next generation sequencing; NTRK+, neurotrophic tropomyosin receptor kinase fusion positive. *One patient had an NTRK+ test before starting treatment (so not included in this table), and treatment information was not available for five patients (so not included in this table). (DOCX) [file pone.0270571.s004.docx]

| **Treatment type** | **Total** | **Line 1** | **Line 2** | **Line 3** | **Line 4** |
| --- | --- | --- | --- | --- | --- |
| **Number of patient‑lines*, n** | 44 | 22 | 12 | 3 | 1 |
| **Chemotherapy, n (%)** | 21 (47.7) | 16 (72.7) | 4 (33.3) | 1 (33.3) | 0 (0.0) |
| **Chemotherapy + hormone therapy, n (%)** | 2 (4.5) | 2 (9.1) | 0 (0.0) | 0 (0.0) | 0 (0.0) |
| **Chemotherapy + targeted therapy, n (%)** | 10 (22.7) | 4 (18.2) | 6 (50.0) | 0 (0.0) | 0 (0.0) |
| **Hormone therapy, n (%)** | 2 (4.5) | 0 (0.0) | 1 (8.3) | 0 (0.0) | 1 (100.0) |
| **Immunotherapy, n (%)** | 1 (2.3) | 0 (0.0) | 0 (0.0) | 1 (33.3) | 0 (0.0) |
| **Targeted therapy, n (%)** | 1 (2.3) | 0 (0.0) | 1 (8.3) | 0 (0.0) | 0 (0.0) |
| **Targeted therapy + hormone therapy, n (%)** | 1 (2.3) | 0 (0.0) | 0 (0.0) | 1 (33.3) | 0 (0.0) |
