# Supplementary material for: Real-world survival outcomes in patients with locally advanced or metastatic NTRK fusion-positive solid tumors receiving standard-of-care therapies other than targeted TRK inhibitors
Source: PLoS One. 2022 Aug 8;17(8):e0270571. doi: 10.1371/journal.pone.0270571 (PMC9359555; doi:10.1371/journal.pone.0270571)
Supplement: S3 Table — Abbreviations: NTRK+, neurotrophic tropomyosin receptor kinase fusion positive. (DOCX) [file pone.0270571.s005.docx]

|  | **None** | **1** | **2** | **3** | **4** | **Unknown** | **Total** |
| --- | --- | --- | --- | --- | --- | --- | --- |
| **Patients, n (% total)** | **1 (3.6)** | **10 (35.7)** | **9 (32.1)** | **2 (7.1)** | **1 (3.6)** | **5 (17.9)** | **28 (100.0)** |
| **Colorectal cancer** |  | 5 (55.6) | 3 (33.3) | 1 (11.1) |  |  | 9 (100.0) |
| **Sarcoma** |  | 1 (16.7) | 1 (16.7) | 1 (16.7) |  | 3 (0.50) | 6 (100.0) |
| **Non-small cell lung cancer** |  | 3 (60.0) | 1 (20.0) |  |  | 1 (20.0) | 5 (100.0) |
| **Salivary gland** | 1 (50.0) |  |  |  |  | 1 (50.0) | 2 (100.0) |
| **Breast** |  |  |  |  | 1 (100.0) |  | 1 (100.0) |
| **Cancer of unknown primary** |  |  | 1 (100.0) |  |  |  | 1 (100.0) |
| **Endometrium** |  |  | 1 (100.0) |  |  |  | 1 (100.0) |
| **Stomach** |  | 1 (100.0) |  |  |  |  | 1 (100.0) |
| **Bile duct** |  |  | 1 (100.0) |  |  |  | 1 (100.0) |
| **Uterus** |  |  | 1 (100.0) |  |  |  | 1 (100.0) |
