# Supplementary material for: Real-world survival outcomes in patients with locally advanced or metastatic NTRK fusion-positive solid tumors receiving standard-of-care therapies other than targeted TRK inhibitors
Source: PLoS One. 2022 Aug 8;17(8):e0270571. doi: 10.1371/journal.pone.0270571 (PMC9359555; doi:10.1371/journal.pone.0270571)
Supplement: S4 Table — Abbreviations: FH-FMI CGDB, Flatiron Health–Foundation Medicine clinicogenomic database; MSI, microsatellite instability; MSI-H, high MSI; MSI-I, intermediate MSI; MSS, microsatellite stability; NA, not available; NTRK-, neurotrophic tropomyosin receptor kinase fusion negative; NTRK+, neurotrophic tropomyosin receptor kinase fusion positive; SMD, standardized mean difference; TMB, tumor mutational burden. *For NTRK+ versus matched NTRK−population. (DOCX) [file pone.0270571.s006.docx]

|  |  | ***NTRK^–^* FH-FMI CGDB (unselected)** | ***NTRK^–^* FH-FMI CGDB (matched)** | ***NTRK*^+^ FH-FMI CGDB** | ***P**** | **SMD** |
| --- | --- | --- | --- | --- | --- | --- |
| **n** |  | **24,903** | **280** | **28** |  |  |
| ***ALK* fusion, n (%)** | Not present | 24,728 (99.3) | 280 (100.0) | 28 (100.0) | NA | <0.001 |
|  | Present | 175 (0.7) | 0 (0.0) | 0 (0.0) |  |  |
| ***ROS1* fusion, n (%)** | Not present | 24,849 (99.8) | 280 (100.0) | 28 (100.0) | NA | <0.001 |
|  | Present | 54 (0.2) | 0 (0.0) | 0 (0.0) |  |  |
| ***RET* fusion, n (%)** | Not present | 24,826 (99.7) | 280 (100.0) | 28 (100.0) | NA | <0.001 |
|  | Present | 77 (0.3) | 0 (0.0) | 0 (0.0) |  |  |
| ***EGFR* L858R, n (%)** | Not present | 24,652 (99.0) | 274 (97.9) | 27 (96.4) | 1 | 0.086 |
|  | Present | 251 (1.0) | 6 (2.1) | 1 (3.6) |  |  |
| ***EGFR* T790M, n (%)** | Not present | 24,833 (99.7) | 280 (100.0) | 28 (100.0) | NA | <0.001 |
|  | Present | 70 (0.3) | 0 (0.0) | 0 (0.0) |  |  |
| ***EGFR* Exon 19, n (%)** | Not present | 24,567 (98.7) | 280 (100.0) | 28 (100.0) | NA | <0.001 |
|  | Present | 336 (1.3) | 0 (0.0) | 0 (0.0) |  |  |
| ***BRAF* V600E, n (%)** | Not present | 24,161 (97.0) | 280 (100.0) | 28 (100.0) | NA | <0.001 |
|  | Present | 742 (3.0) | 0 (0.0) | 0 (0.0) |  |  |
| ***BRAF* V600K, n (%)** | Not present | 24,829 (99.7) | 280 (100.0) | 28 (100.0) | NA | <0.001 |
|  | Present | 74 (0.3) | 0 (0.0) | 0 (0.0) |  |  |
| ***MET* Exon 14, n (%)** | Not present | 24,867 (99.9) | 280 (100.0) | 28 (100.0) | NA | <0.001 |
|  | Present | 36 (0.1) | 0 (0.0) | 0 (0.0) |  |  |
| ***KRAS* G12, n (%)** | Not present | 19,861 (79.8) | 273 (97.5) | 27 (96.4) | 1 | 0.062 |
|  | Present | 5042 (20.2) | 7 (2.5) | 1 (3.6) |  |  |
| ***KRAS* G13, n (%)** | Not present | 24,272 (97.5) | 280 (100.0) | 28 (100.0) | NA | <0.001 |
|  | Present | 631 (2.5) | 0 (0.0) | 0 (0.0) |  |  |
| **MSI status, n (%)** | MSI-H | 318 ( 1.3) | 52 (18.6) | 5 (17.9) | 0.989 | 0.069 |
|  | MSI-I | 69 (0.3) | 0 (0.0) | 0 (0.0) |  |  |
|  | MSS | 17,759 (71.3) | 126 (45.0) | 12 (42.9) |  |  |
|  | Not measured | 4,909 (19.7) | 91 (32.5) | 10 (35.7) |  |  |
|  | MSI Unknown | 1,848 (7.4) | 11 (3.9) | 1 (3.6) |  |  |
| **TMB status, n (%)** | High | 1,362 (5.5) | 54 (19.3) | 6 (21.4) | 0.989 | 0.113 |
|  | Intermediate | 6,003 (24.1) | 50 (17.9) | 5 (17.9) |  |  |
|  | Low | 9,664 (38.8) | 62 (22.1) | 5 (17.9) |  |  |
|  | Not measured | 6,101 (24.5) | 104 (37.1) | 11 (39.3) |  |  |
|  | Unknown | 1,773 (7.1) | 10 (3.6) | 1 (3.6) |  |  |
