# Supplementary material for: Real-world survival outcomes in patients with locally advanced or metastatic NTRK fusion-positive solid tumors receiving standard-of-care therapies other than targeted TRK inhibitors
Source: PLoS One. 2022 Aug 8;17(8):e0270571. doi: 10.1371/journal.pone.0270571 (PMC9359555; doi:10.1371/journal.pone.0270571)
Supplement: S5 Table — Abbreviations: FH-FMI CGDB, Flatiron Health–Foundation Medicine clinicogenomic database; NTRK-, neurotrophic tropomyosin receptor kinase fusion negative; NTRK+, neurotrophic tropomyosin receptor kinase fusion positive. (DOCX) [file pone.0270571.s007.docx]

|  | **Overall  FH-FMI CGDB** | ***NTRK^–^*  FH-FMI CGDB (unselected)** | ***NTRK^+^* FH-FMI CGDB** | ***P*** |
| --- | --- | --- | --- | --- |
| **Patients, n (%)** | **6,070** | **6,065** | **5** |  |
| **History of smoking** | 5,050 (83.2) | 5,045 (83.2) | 5 (100.0) | 0.603 |
| **No history of smoking** | 999 (16.5) | 999 (16.5) | 0 (0.0) |  |
| **Unknown/not documented** | 21 (0.3) | 21 (0.3) | 0 (0.0) |  |
