# Supplementary material for: Real-world survival outcomes in patients with locally advanced or metastatic NTRK fusion-positive solid tumors receiving standard-of-care therapies other than targeted TRK inhibitors
Source: PLoS One. 2022 Aug 8;17(8):e0270571. doi: 10.1371/journal.pone.0270571 (PMC9359555; doi:10.1371/journal.pone.0270571)
Supplement: S6 Table — Abbreviations: CNS, central nervous system; FH-FMI CGDB, Flatiron Health–Foundation Medicine clinicogenomic database; GI, gastrointestinal; GIST, gastrointestinal stromal tumor; neuro, neuroendocrine; NK T cells, natural killer T cells; NTRK-, neurotrophic tropomyosin receptor kinase fusion negative. The shades of orange represent the three largest groups in each tumor type (darker shades show the largest groups). (DOCX) [file pone.0270571.s008.docx]

|  | **None** | **1** | **2** | **3** | **4** | **5+** | **Unknown** | **Total** |
| --- | --- | --- | --- | --- | --- | --- | --- | --- |
| **Patients, n (% of total)** | **2,902 (11.7)** | **9,683 (38.9)** | **4,361 (17.5)** | **2,273 (9.1)** | **1,077 (4.3)** | **1,469 (6.0)** | **3,138 (12.6)** | **24,903 (100.0)** |
| **Adenoid cystic carcinoma** | 13 (18.3) | 16 (22.5) | 5 (7.0) |  |  |  | 37 (52.1) | 71 (100.0) |
| **Adrenal gland** | 7 (20.6) | 13 (38.2) | 4 (11.8) | 1 (2.9) |  | 1 (2.9) | 8 (23.5) | 34 (100.0) |
| **Angiosarcoma** | 5 (17.2) | 11 (37.9) | 4 (13.8) | 3 (10.3) | 2 (6.9) | 1 (3.4) | 3 (10.3) | 29 (100.0) |
| **Anus** | 2 (1.9) | 38 (36.9) | 37 (35.9) | 9 (8.7) | 7 (6.8) | 1 (1.0) | 9 (8.7) | 103 (100.0) |
| **Appendix** | 12 (0.1) | 40 (38.8) | 26 (25.2) | 13 (10.8) | 5 (4.2) | 5 (4.2) | 19 (15.8) | 120 (100.0) |
| **Biliary** | 23 (8.0) | 123 (43.0) | 68 (23.8) | 22 (7.7) | 5 (1.7) | 2 (0.7) | 43 (15.0) | 286 (100.0) |
| **Bladder** | 59 (10.3) | 249 (43.5) | 132 (23.0) | 50 (8.7) | 12 (2.1) | 3 (0.5) | 68 (11.9) | 573 (100.0) |
| **Bone sarcoma** | 3 (7.9) | 13 (34.2) | 7 (18.4) | 1 (2.6) | 3 (7.9) |  | 11 (2.9) | 38 (100.0) |
| **Breast** | 141 (4.7) | 613 (20.6) | 536 (18.1) | 495 (16.7) | 311 (10.5) | 641 (21.6) | 232 (7.8) | 2,969 (100.0) |
| **Carcinoid** | 6 (8.3) | 21 (29.2) | 16 (22.2) | 2 (2.8) | 2 (2.8) |  | 25 (34.7) | 72 (100.0) |
| **Cervix** | 7 (4.2) | 53 (31.5) | 43 (25.6) | 26 (15.5) | 10 (6.0) | 7 (4.2) | 22 (13.1) | 168 (100.0) |
| **Cholangiocarcinoma** | 34 (8.1) | 194 (46.4) | 76 (18.2) | 30 (7.2) | 9 (2.2) | 5 (1.2) | 70 (16.7) | 418 (100.0) |
| **Chondrosarcoma** | 5 (20.8) | 4 (16.7) | 1 (4.2) |  |  | 1 (41.7) | 13 (54.2) | 24 (100.0) |
| **CNS non-glioma** | 1 (16.7) | 2 (33.3) |  |  |  | 1 (16.7) | 2 (33.3) | 6 (100.0) |
| **Colorectal cancer** | 264 (6.3) | 1,606 (38.3) | 868 (20.7) | 476 (11.3) | 264 (6.3) | 321 (7.6) | 398 (9.5) | 4,197 (100.0) |
| **Endocrine-neuro** | 11 (12.5) | 29 (33.0) | 16 (18.2) | 3 (3.4) | 1 (1.1) |  | 28 (31.8) | 88 (100.0) |
| **Endometrial** | 25 (10.5) | 75 (31.6) | 60 (25.3) | 24 (10.1) | 12 (5.1) | 11 (4.6) | 30 (12.7) | 237 (100.0) |
| **Esophagus** | 50 (5.2) | 437 (45.1) | 231 (23.9) | 107 (11.1) | 25 (2.6) | 30 (3.1) | 88 (9.1) | 968 (100.0) |
| **Ewing sarcoma** |  | 2 (28.6) | 3 (42.9) | 1 (14.3) |  | 1 (14.3) |  | 7 (100.0) |
| **Eye** |  | 1 (33.3) |  |  |  |  | 2 (66.7) | 3 (100.0) |
| **Fallopian tube** |  | 2 (40.0) | 2 (40.0) | 1 (20.0) |  |  |  | 5 (100.0) |
| **Female genital** | 1 (2.6) | 19 (48.7) | 8 (20.5) | 4 (10.3) | 1 (2.6) |  | 6 (15.4) | 39 (100.0) |
| **Female-neuro** | 1 (5.6) | 7 (38.9) | 4 (22.2) | 2 (11.1) | 2 (11.1) | 1 (5.6) | 1 (5.6) | 18 (100.0) |
| **Germ cell** | 3 (10.3) | 13 (44.8) | 9 (31.0) |  | 1 (3.4) |  | 3 (10.3) | 29 (100.0) |
| **GI-neuro** | 7 (10.3) | 29 (42.6) | 15 (22.1) | 2 (2.9) | 2 (2.9) |  | 13 (19.1) | 68 (100.0) |
| **GIST** | 5 (6.0) | 48 (57.8) | 15 (18.1) | 5 (6.0) | 3 (3.6) | 4 (4.8) | 3 (3.6) | 83 (100.0) |
| **Glioma** | 1 (14.3) | 2 (28.6) | 1 (14.3) | 1 (14.3) |  | 1 (14.3) | 1 (14.3) | 7 (100.0) |
| **Glomus** | 1 (100.0) |  |  |  |  |  |  | 1 (100.0) |
| **Head and neck** | 30 (8.6) | 109 (31.3) | 83 (23.9) | 56 (16.1) | 24 (6.9) | 11 (3.2) | 35 (10.1) | 348 (100.0) |
| **Head and neck-neuro** | 1 (20.0) | 2 (40.0) |  |  |  | 1 (20.0) | 1 (20.0) | 5 (100.0) |
| **Histiocytosis** |  | 2 (33.3) | 2 (33.3) | 1 (16.7) |  | 1 (16.7) |  | 6 (100.0) |
| **Kaposi sarcoma** | 1 (50.0) |  |  |  |  |  | 1 (50.0) | 2 (100.0) |
| **Kidney** | 66 (12.3) | 214 (40.0) | 92 (17.2) | 50 (9.3) | 24 (4.5) | 27 (5.0) | 62 (11.6) | 535 (100.0) |
| **Leiomyosarcoma** | 17 (11.0) | 62 (40.3) | 25 (16.2) | 9 (5.8) | 7 (4.5) | 3 (1.9) | 31 (20.1) | 154 (100.0) |
| **Liver** | 31 (20.7) | 69 (46.0) | 18 (12.0) | 4 (2.7) | 2 (1.3) |  | 26 (17.3) | 150 (100.0) |
| **Non-small cell lung cancer** | 1,257 (20.7) | 2,640 (43.5) | 778 (12.8) | 349 (5.8) | 123 (2.0) | 112 (1.8) | 805 (13.3) | 6,064 (100.0) |
| **Lung sarcoma** |  |  | 1 (100.0) |  |  |  |  | 1 (100.0) |
| **Male genital** | 1 (12.5) | 1 (12.5) | 3 (37.5) | 2 (25.0) |  |  | 1 (12.5) | 8 (100.0) |
| **Male-neuro** | 1 (7.1) | 5 (35.7) | 4 (28.6) |  |  | 1 (7.1) | 3 (2.1) | 14 (100.0) |
| **Melanoma** | 173 (20.0) | 394 (45.6) | 100 (11.6) | 27 (3.1) | 14 (1.6) | 9 (1.0) | 147 (17.0) | 864 (100.0) |
| **Mesothelioma** | 12 (9.4) | 61 (48.0) | 31 (24.4) | 5 (3.9) | 2 (1.6) | 2 (1.6) | 14 (11.0) | 127 (100.0) |
| **Myelodysplastic syndrome** |  |  | 1 (100.0) |  |  |  |  | 1 (100.0) |
| **Myeloproliferative neoplasm** | 1 (100.0) |  |  |  |  |  |  | 1 (100.0) |
| **NK T cell neoplasm** |  |  | 1 (50.0) |  |  |  | 1 (50.0) | 2 (100.0) |
| **Non-Hodgkin lymphoma** | 1 (50.0) |  |  |  |  |  | 1 (50.0) | 2 (100.0) |
| **Ovary** | 9 (8.7) | 39 (37.5) | 26 (25.0) | 3 (2.9) | 4 (3.8) | 8 (7.7) | 15 (14.4) | 104 (100.0) |
| **Pancreas** | 106 (6.2) | 721 (42.4) | 379 (22.3) | 177 (10.4) | 78 (4.6) | 34 (20.0) | 206 (12.1) | 1,701 (100.0) |
| **Peripheral nervous system** | 3 (37.5) | 1 (12.5) |  |  | 1 (12.5) |  | 3 (37.5) | 8 (100.0) |
| **Peritoneum** | 1 (14.3) | 2 (28.6) |  | 2 (28.6) | 1 (14.3) |  | 1 (14.3) | 7 (100.0) |
| **Placenta** |  | 1 (100.0) |  |  |  |  |  | 1 (100.0) |
| **Prostate** | 51 (5.8) | 310 (35.1) | 147 (16.7) | 100 (11.3) | 57 (6.5) | 158 (17.9) | 59 (6.7) | 882 (100.0) |
| **Rhabdomyosarcoma** |  | 3 (17.6) | 6 (35.3) | 2 (11.8) |  | 1 (5.9) | 5 (29.4) | 17 (100.0) |
| **Salivary gland** | 12 (32.4) | 8 (21.6) | 3 (8.1) | 2 (5.4) | 1 (2.7) | 2 (5.4) | 9 (24.3) | 37 (100.0) |
| **Skin** | 15 (19.7) | 24 (31.6) | 12 (15.8) | 4 (5.3) | 3 (3.9) | 3 (3.9) | 15 (19.7) | 76 (100.0) |
| **Skin sarcoma** | 1 (100.0) |  |  |  |  |  |  | 1 (100.0) |
| **Skin-neuro** | 6 (19.4) | 12 (38.7) | 6 (19.4) | 4 (12.9) |  | 1 (3.2) | 2 (6.5) | 31 (100.0) |
| **Small cell** | 7 (10.0) | 31 (43.7) | 15 (21.1) | 9 (12.7) | 4 (5.6) | 2 (2.8) | 3 (4.2) | 71 (100.0) |
| **Small intestine** | 13 (9.4) | 53 (38.4) | 31 (22.5) | 13 (9.4) | 3 (2.2) | 4 (2.9) | 21 (15.2) | 138 (100.0) |
| **Soft tissue sarcoma** | 47 (16.7) | 100 (35.6) | 37 (13.2) | 23 (8.2) | 5 (1.8) | 3 (1.1) | 66 (23.5) | 281 (100.0) |
| **Solitary fibrous tumor** | 3 (27.3) | 3 (27.3) | 2 (18.2) | 1 (9.1) |  |  | 2 (18.2) | 11 (100.0) |
| **Stomach** | 28 (5.4) | 257 (49.8) | 99 (19.2) | 39 (7.6) | 15 (2.9) | 18 (3.5) | 60 (11.6) | 516 (100.0) |
| **Testis** |  |  |  |  |  |  | 1 (100.0) | 1 (100.0) |
| **Thymus** | 2 (6.9) | 14 (48.3) | 5 (17.2) |  |  |  | 8 (27.6) | 29 (100.0) |
| **Thymus thymoma** | 2 (12.5) | 9 (56.3) | 3 (18.8) |  | 1 (6.3) |  | 1 (6.3) | 16 (100.0) |
| **Thyroid** | 28 (23.3) | 33 (27.5) | 7 (5.8) | 2 (1.7) |  |  | 50 (41.7) | 120 (100.0) |
| **Unclassified** |  |  |  | 1 (50.0) |  |  | 1 (50.0) | 2 (100.0) |
| **Underspecified** | 19 (17.8) | 53 (49.5) | 10 (9.3) | 4 (3.7) | 1 (0.9) |  | 20 (18.7) | 107 (100.0) |
| **Cancer of unknown primary** | 244 (16.5) | 659 (44.6) | 176 (11.9) | 78 (5.3) | 25 (1.7) | 26 (1.8) | 269 (18.2) | 1,477 (100.0) |
| **Unknown primary-neuro** | 15 (8.5) | 78 (44.1) | 31 (17.5) | 9 (5.1) | 2 (1.1) | 4 (2.3) | 38 (21.5) | 177 (100.0) |
| **Urinary** | 7 (10.0) | 25 (35.7) | 21 (30.0) | 7 (10.0) | 2 (28.6) | 1 (1.4) | 7 (10.0) | 70 (100.0) |
| **Urinary-neuro** | 1 (10.0) | 3 (30.0) | 1 (10.0) | 2 (20.0) | 1 (10.0) |  | 2 (20.0) | 10 (100.0) |
| **Uterus** | 2 (38.5) | 21 (40.4) | 13 (25.0) | 7 (13.5) |  |  | 9 (17.3) | 52 (100.0) |
| **Uterus sarcoma** | 1 (6.7) | 4 (26.7) | 5 (33.3) | 3 (20.0) |  |  | 2 (13.3) | 15 (100.0) |
