# Supplementary material for: Real-world survival outcomes in patients with locally advanced or metastatic NTRK fusion-positive solid tumors receiving standard-of-care therapies other than targeted TRK inhibitors
Source: PLoS One. 2022 Aug 8;17(8):e0270571. doi: 10.1371/journal.pone.0270571 (PMC9359555; doi:10.1371/journal.pone.0270571)
Supplement: S7 Table — Abbreviations: CNS, central nervous system; FH-FMI CGDB, Flatiron Health–Foundation Medicine clinicogenomic database; GI, gastrointestinal; GIST, gastrointestinal stromal tumor; neuro, neuroendocrine; NK T cells, natural killer T cells; NTRK-, neurotrophic tropomyosin receptor kinase fusion negative; NTRK+, neurotrophic tropomyosin receptor kinase fusion positive; OS, overall survival. Green: tumor types with median OS >10 months; Red: tumor types with median OS <5 months; Gray: tumor types with unavailable median OS. (DOCX) [file pone.0270571.s009.docx]

| **Tumor type, N (%)** | ***NTRK^–^*  FH-FMI CGDB (unselected)** | **Median OS, months** | **In *NTRK*^+^  FH-FMI CGDB cohort** |
| --- | --- | --- | --- |
|  | **24903** |  |  |
| **Non-small cell lung cancer** | 6064 (24.4) | 8.41 | Yes |
| **Colorectal cancer** | 4197 (16.9) | 16.69 | Yes |
| **Breast** | 2969 (11.9) | 11.37 | Yes |
| **Pancreas** | 1701 (6.8) | 5.52 | — |
| **Cancer of unknown primary** | 1477 (5.9) | 5.82 | Yes |
| **Esophagus** | 968 (3.9) | 7.82 | — |
| **Prostate** | 882 (3.5) | 21.85 | — |
| **Melanoma** | 864 (3.5) | 13.14 | — |
| **Bladder** | 573 (2.3) | 6.41 | — |
| **Kidney** | 535 (2.1) | 10.02 | — |
| **Stomach** | 516 (2.1) | 7.75 | Yes |
| **Cholangiocarcinoma** | 418 (1.7) | 6.51 | — |
| **Head and neck** | 348 (1.4) | 7.59 | — |
| **Biliary** | 286 (1.1) | 5.49 | Yes |
| **Soft tissue sarcoma** | 281 (1.1) | 9.82 | Yes |
| **Endometrial** | 237 (1.0) | 9.86 | Yes |
| **Unknown primary-neuro** | 177 (0.7) | 5.39 | — |
| **Cervix** | 168 (0.7) | 7.95 | — |
| **Leiomyosarcoma** | 154 (0.6) | 12.22 | — |
| **Liver** | 150 (0.6) | 6.01 | — |
| **Small intestine** | 138 (0.6) | 9.89 | — |
| **Mesothelioma** | 127 (0.5) | 7.20 | — |
| **Appendix** | 120 (0.5) | 16.00 | — |
| **Thyroid** | 120 (0.5) | 9.79 | — |
| **Underspecified** | 107 (0.4) | 4.73 | — |
| **Ovary** | 104 (0.4) | 8.64 | — |
| **Anus** | 103 (0.4) | 9.76 | — |
| **Endocrine-neuro** | 88 (0.4) | 6.70 | — |
| **GIST** | 83 (0.3) | 20.27 | — |
| **Skin** | 76 (0.3) | 7.85 | — |
| **Carcinoid** | 72 (0.3) | 26.61 | — |
| **Adenoid cystic carcinoma** | 71 (0.3) | 16.92 | — |
| **Small cell** | 71 (0.3) | 8.21 | — |
| **Urinary** | 70 (0.3) | 9.96 | — |
| **GI-neuro** | 68 (0.3) | 7.92 | — |
| **Uterus** | 52 (0.2) | 4.63 | Yes |
| **Female genital** | 39 (0.2) | 6.77 | — |
| **Bone sarcoma** | 38 (0.2) | 13.90 | — |
| **Salivary gland** | 37 (0.1) | 11.20 | Yes |
| **Adrenal gland** | 34 (0.1) | 9.23 | — |
| **Skin-neuro** | 31 (0.1) | 9.17 | — |
| **Thymus** | 29 (0.1) | 18.37 | — |
| **Germ cell** | 29 (0.1) | 10.81 | — |
| **Angiosarcoma** | 29 (0.1) | 8.61 | — |
| **Chondrosarcoma** | 24 (0.1) | 6.51 | — |
| **Female-neuro** | 18 (0.1) | 9.17 | — |
| **Rhabdomyosarcoma** | 17 (0.1) | 11.76 | — |
| **Thymus thymoma** | 16 (0.1) | 14.13 | — |
| **Uterus sarcoma** | 15 (0.1) | 4.73 | — |
| **Male-neuro** | 14 (0.1) | 6.97 | — |
| **Solitary fibrous tumor** | 11 (0.0) | 33.28 | — |
| **Urinary-neuro** | 10 (0.0) | 9.89 | — |
| **Peripheral nervous system** | 8 (0.0) | 16.36 | — |
| **Male genital** | 8 (0.0) | 2.12 | — |
| **Ewing sarcoma** | 7 (0.0) | 11.66 | — |
| **Peritoneum** | 7 (0.0) | 6.21 | — |
| **Glioma** | 7 (0.0) | 3.24 | — |
| **CNS non-glioma** | 6 (0.0) | 17.10 | — |
| **Histiocytosis** | 6 (0.0) | 6.70 | — |
| **Head and neck-neuro** | 5 (0.0) | 28.35 | — |
| **Fallopian tube** | 5 (0.0) | 1.45 | — |
| **Eye** | 3 (0.0) | 3.42 | — |
| **Non-Hodgkin lymphoma** | 2 (0.0) | 8.23 | — |
| **Kaposi sarcoma** | 2 (0.0) | 2.23 | — |
| **Unclassified** | 2 (0.0) | 1.74 | — |
| **NK T cell neoplasm** | 2 (0.0) | NA | — |
| **Glomus** | 1 (0.0) | 42.09 | — |
| **Lung sarcoma** | 1 (0.0) | 34.00 | — |
| **Skin sarcoma** | 1 (0.0) | 10.35 | — |
| **Myeloproliferative neoplasm** | 1 (0.0) | 0.76 | — |
| **Myelodysplastic syndrome** | 1 (0.0) | NA | — |
| **Placenta** | 1 (0.0) | NA | — |
| **Testis** | 1 (0.0) | NA | — |
